# 

**Published:** 1879-03

**Authors:** 


THE BUFFALO

### MEDICAL AND SURGICAL

### JOURNAL.

VOL. XVIII. MARCH, 1879. NO. 8.

